# Physicochemical properties of AH plus bioceramic sealer, Bio-C Sealer, and ADseal root canal sealer

**DOI:** 10.1186/s13005-023-00403-z

**Published:** 2024-01-03

**Authors:** Tamer M. Hamdy, Manar M. Galal, Amira Galal Ismail, Shehabeldin Saber

**Affiliations:** 1 Restorative and Dental Materials Department, Oral and Dental Research Institute, National Research Centre (NRC), Giza, Dokki, 12622 Egypt; 2 Endodontic Department, Faculty of Dentistry, The British University in Egypt, Cairo, 11841 Egypt; 3 Center for Innovative Dental Sciences, The British University in Egypt, Cairo, 11841 Egypt; 4 Endodontic Department, Faculty of Dentistry, Ain Shams University, Cairo, 11566 Egypt

**Keywords:** Bioceramic Sealer, AH Plus Bioceramic Sealer, Bio-C Sealer, ADseal, Solubility, pH change, Calcium ions release, Film thickness

## Abstract

**Background:**

The aim of this study was to evaluate the physicochemical properties of two newly introduced premixed calcium silicate-based root canal sealers (AH Plus Bioceramic Sealer and Bio-C Sealer) compared to a resin-based root canal sealer (ADseal root canal sealer).

**Methods:**

Solubility, pH analysis, calcium ion release, and film thickness of each sealer were evaluated following ISO guidelines. The data were examined using the two-way ANOVA test. Furthermore, X-ray diffraction (XRD) examination was performed to investigate the crystalline phase of each type of sealer. X-ray fluorescence (XRF) analysis was done for the chemical elemental analysis of each sealer.

**Results:**

The least film thickness, highest alkalinity, and highest calcium ion release were all displayed by AH Plus Bioceramic Sealer. High solubility, high alkalinity, intermediate calcium ion release, and intermediate film thickness were all displayed by Bio-C Sealer. While ADseal root canal sealer displayed the greatest film thickness, least solubility, alkalinity, and calcium ion release.

**Conclusions:**

Both AH Plus Bioceramic Sealer and Bio-C Sealer represented adequate properties to be considered a good sealer that could be used as a potential alternative to resin-based root canal sealers.

## Background

The principal intention of root canal sealers is to achieve hermetically sealed root canals, possess healthy periapical tissue, and avoid reinfection of the root canal [1]. Consequently, ideal root canal sealers should be capable of preventing leakage, and minimizing the possibility of bacterial invasion [2, 3]. Improvement in root canal materials, together with advancement in endodontic file design and metallurgy, is required continuously to develop a proper endodontic treatment [4–7]. A great diversity of root canal sealers is available commercially; they differ in their biological, thermal, chemical, and physical properties [8, 9].

Insolubility is the main prerequisite for ideal root canal sealers to offer perfect sealing ability [1]. Furthermore, both the alkalinity and bioactive properties of the root canal sealer materials are significant factors that provide a great chance for tissue healing and remineralization [10, 11]. Film thickness is an important feature of a root canal sealer to ensure the perfect sealing ability of the root canal systems. Also, it affects the handling performance of the sealers [12].

At present, resin-based root canal sealer serves as the most commonly used root canal sealer materials. It provides appropriate physical properties including solubility and alkalinity, and an adequate apical seal [2, 13]. However, there is no chemical bond between the tooth structure and the root canal sealer [14]. Hence, there has been a continuous request for alternative sealers that are capable of bonding to the root canal wall.

The term “ceramic materials” refers to an inorganic, non-metallic, frequently crystalline oxide, nitride, or carbide material [15]. Biocompatible ceramics refer to a type of ceramic materials used for specific biological or physiological functions [16]. According to the application, bioceramics can directly interact with the adjacent tissue, either promoting tissue growth or triggering new tissue regeneration [17]. The advent of bioceramic materials has recently been conducted as a successful root canal filling material [18, 19]. Bioceramic materials can be categorized into bioinert or bioactive materials based on their interaction with the adjacent tissue [20, 21]. Zirconia is a bioinert material; it refers to artificially produced crystals of zirconium oxide (ZrO_2_), Zirconium and hafnium continuously arise together as naturally occurring minerals. Zirconium arises mainly as a silicate in zircon (ZrSiO_4_) and as an oxide in baddeleyite [22]. On the other hand, bioactive materials, such as hydroxyapatite (HA), calcium silicates, and calcium phosphates, can actively undergo interactions at the interface with the surrounding tissues to encourage precipitation of HA layer, promoting a chemical bond between the bioactive sealer and dentin [10, 23–25].

Bioceramic-based sealers are divided according to their main chemical composition into two main categories: calcium silicate-based and calcium phosphate-based sealers. Other fillers can be added to improve the physicochemical features of root canal sealers [21, 26, 27]. Moreover, enhancement of bioactivity through promotion of HA formation and deposition of an apatite-like layer facilitates the bond between the dentin and root canal sealers at the interface [23, 28–31].

Recent premixed calcium silicate-based root canal sealers (AH Plus Bioceramic Sealer and Bio-C Sealer) have claimed to provide favorable handling characteristics compared to conventional resin-based root canal sealer that have already demonstrated considerable clinical success [32]. Moreover, they are claimed to provide the benefits of a bioceramic formulation that induces the formation of mineralized tissue by releasing calcium ions and enhancing alkalinity.

Therefore, this laboratory study aimed to examine the physicochemical properties (solubility, pH analysis, calcium release, and film thickness) of newly introduced premixed calcium silicate-based root canal sealers (AH Plus Bioceramic Sealer and Bio-C Sealer) in comparison to ADseal root canal resin-based sealer. The null hypothesis was that there is no difference between the new premixed calcium silicate-based root canal sealers (AH Plus Bioceramic Sealer and Bio-C Sealer) and ADseal root canal sealer with respect to solubility, pH analysis, calcium ion release, and film thickness.

## Methods

The study was approved by the Medical Research Ethical Committee (MREC) of the National Research Centre (NRC), Cairo, Egypt (approval number for the study: 3,911,911,022). The root canal sealers examined in the current study are shown in Table 1.

**Table 1:** Chemical composition of the root canal sealers used in the study

Root canal sealers	Manufacturer	Chemical composition*
AH Plus Bioceramic Sealer (premixed calcium silicate-based root canal sealers)	Dentsply, De-Trey Konstanz, Germany	Zirconium dioxide (50–75%), tricalcium silicate (5–15%), dimethyl sulfoxide (10–30%), lithium carbonate (< 0.5%), thickening agent (< 6%)
Bio-C Sealer (premixed calcium silicate-based root canal sealers)	Angelus, Londrina, PR, Brazil	Calcium and magnesium silicate, calcium sulfate, potassium sulfate, zirconium oxide, silicon dioxide and dispersing agent
ADseal root canal sealer (resin-based sealer)	Metabiomed, Cheongju, Korea	Poly(1,4-butanediol) bis(4-aminobenzoate) (30–40%), Bisphenol A diglycidyl ether–bisphenol A copolymer (20–30%), 2-Hydroxyethyl salicylate (15–20%), Triethanolamine (< 10), calcium oxide (< 5%).

* The chemical compositional concentration data were extracted from the respective Material Safety Data Sheets (MSDS), if available and presented as a percentage by weight (WT%)

### Calculation of the sample size

The sample size calculation was performed according to G*Power software version 3.1.9.2 (Heinrich Heine University, Dusseldorf, Germany) at a significance level of *p* 0.05 for evaluation of solubility, pH change, calcium ion release, and film thickness [33–35]. The indicated samples were 10 for each group.

### Solubility test

The solubility test was carried out in accordance with the International Standard Organization (ISO 6876:2012) [29]. Teflon moulds measuring 1.5 mm in height and 7.75 mm in inner diameter were fabricated and fully filled with each root canal sealer to obtain a disc-shaped specimen [31, 34, 36]. Each specimen was incubated at 37 °C for 24 h in 95% relative humidity to set, the specimens were removed after setting from the mould and weighed (M1) using an analytical balance (Adam Equipment 4-digit precision weighing balance, UK) with an accuracy of 0.001 g [36]. Then, specimens of each root canal sealer were hanged using a nylon thread in a closed plastic flask containing 7.5 mL of distilled water and stored for two successive time intervals, which are 7 days and 14 days, in the incubator at 37 °C and 95% relative humidity. The specimens were removed from the incubator, dried with absorbent paper, and placed in a dehumidifying chamber for 24 h [31, 37]. Then the specimens were re-weighed (M2). Mass loss was expressed as a percentage of the original mass. The percentage of root canal sealers solubility was calculated using the formula:

(M1-M2)/M1 × 100% [31, 37, 38]

where M1 is the initial mass and M2 is the final mass of the specimens [33, 34].

### pH analysis

Each type of root canal sealer was inserted into polytetrafluoroethylene tubes to obtain discs with a 5 mm diameter and 2 mm thickness [35]. After the sealer setting, each specimen was immersed into closed flask containing 10 mL of distilled water at an initial pH of 7 and a temperature of 25 °C. Then the specimens were stored in an incubator at 37 °C and 95% relative humidity for 7 and 14 days. The calibration of the pH meter (Jen-way 3510 bench pH meter, UK) was performed with a standard solution at pH 4.0 and 7.0 at a constant temperature of 25 °C. The pH of the solution was measured immediately after 7 and 14 days of immersion [28, 31].

### Calcium ions release

The previous solutions were used to measure the release of calcium ions using optical emission spectroscopy (ICP-OES) (Ultima 2 ICP, Horiba, USA). The cumulative amounts of the released calcium ions from each sealer were measured after 7 and 14 days, respectively (mg/L). After each time intervals, 10 ml of the immersion solution of each specimen was withdrawn by a plastic syringe and forced into a plastic falcon tube for ICP analysis with spectral range between 160 and 800 nm. The sample solution from each falcon tube was nebulized. Following calibration, the amount of each element present in solution was determined by analyzing the intensity of the radiation emitted at the specific elemental frequency to detect the released calcium ions [28, 29, 35].

### Film thickness

The film thickness of each sealer was investigated according to the International Standard Organization (ISO) 6876:2012 instructions [39]. Two pieces of flat glass plates (5 mm in thickness, 200 × 10 mm surface area) were placed over one another. Total thickness was measured using an electronic digital caliper (Digital Vernier Caliper, Mitutoyo, Japan). Each endodontic root canal sealer was prepared according to the manufacturers’ instructions. After mixing, 0.5 ml of each sealer was transferred immediately onto the lower glass plate and was covered by the upper glass plate. A 150 N load weight was vertically applied for 180 ± 10 sseconds on the upper glass plate. The total thickness of the plates, including the sealer, was measured using a digital caliper after 10 min from the mixing time (7 min from the time of applying the force). The value of the film thickness was obtained by subtracting the previous reading from the total thickness of the glass plates. The mean value of the film thickness for each sealer specimens was recorded by repeating the reading three times and calculating the average value.

### XRD investigation

The crystalline structure and chemical composition were investigated by an X-ray powder diffraction analysis (XRD) system (Bruker-AXS D8 X-ray diffractometer, Germany). After setting the freshly mixed sealer specimens, the disc specimens were ground progressively by an agate mortar and pestle till a finer powder was obtained. An amount of 0.2 g of powder from each group was placed between two pieces of magic tape on the X-ray diffractometer. The test was conducted in continuous mode at an angle 2 range of (0–60°) with a scanning rate of 4°/minute under 30.0 mA at 40.0 kV. The attained XRD patterns were interpreted using the model pattern on the Joint Committee on Powder Diffraction Standard (JCPDS) databases [40].

### XRF investigation

The quantitative chemical elemental analysis of the tested sealers was performed using XRF analysis (X-MET3000TXR, Oxford Instruments GmbH Co., Borsigstrasse, Germany). The powder from each group were obtained as previously described in XRD investigation. On micro-carry paper, powder was loaded. The miniature X-ray tubes with Rh anodes in the XRF spectrometer were used, and it was run at 50 kV and 2 mA. The diode detectors were used to obtain the XRF patterns for the sealers, and an XRF analyzer was used to analyze them [41].

### Statistical analysis

According to the normality test performed using (Kolmogorov-Smirnov and Shapiro-Wilk tests). The data of solubility %, pH analysis, calcium ions release, and film thickness were statistically analyzed by the two-way ANOVA and Tukey’s post hoc tests using SPSS software 16.0 statistical software (SPSS Inc., Chicago, IL, USA). The significance level was set at *P*-value ≤ 0.05.

## Results

### Solubility test

Table 2 illustrates the comparison between the mean values of the solubility percentage of the tested sealers at different immersion times. There were significant differences in the solubility between the three tested sealers after 7 days (*P*-value = 0.0001*). A similar finding was detected after 14 days (*P*-value = 0.0001*). The ADseal showed the least solubility, while the Bio-C Sealer displayed the highest solubility. Meanwhile, the AH Plus Bioceramic Sealer showed intermediate results. Within each material, the solubility increased significantly with time (from day 7 to day 14; *P*-value = 0.0001*).

**Table 2:** The mean values of solubility % of the tested sealers at different immersion times (n = 10 per sealer)

Sealer	ADseal sealer (control)	AH Plus Bioceramic Sealer	Bio-C Sealer	*P*-value
**Solubility % (day 7)**	1^aI^ ± 0.1	2.3 ^bI^±0.08	2.8 ^cI^±0.1	0.0001*
**Solubility % (day 14)**	1.21^aII^ ± 0.5	2.61^bII^ ± 0.08	3.00 ^cII^±0.08	0.0001*
***P***-value	0.0001*	0.0001*	0.0001*	

Means with different small letters in the same row indicate a significant difference, while means with different capital Roman letters in the same column also indicate a significant difference. *Indicates a statistically significant difference (*P*-value < 0.05)

### pH analysis

The result of the pH analysis for all tested sealers in all time periods is shown in Table 3. There were significant differences in the pH values between the tested sealers after 7 days; the AH Plus Bioceramic Sealer showed the highest pH value, followed by the Bio-C Sealer, while the sealer with the least pH was the ADseal root canal sealer. Similarly, after 14 days, there was a significant difference in the pH values between the sealers in the same order as the one that occurred: AH Plus Bioceramic sealer ˃ Bio-C Sealer ˃ ADseal root canal sealer.

The comparison of the pH analysis within each sealer separately at the two different time intervals (7 days versus 14 days) showed that the pH values increased significantly in the AH Plus Bioceramic sealer as well as in the Bio-C Sealer. On the other hand, the pH of the ADseal root canal sealer remained constant from 7 to 14 days with no significant change by time.

**Table 3:** The mean values of pH analysis of all tested sealers at different immersion times (n = 10 per sealer)

Sealers pH	AD seal Sealer (control)	AH Plus Bioceramic Sealer	Bio-C Sealer	*P*-value
**7 days**	8.5 ^aI^±0.1	10.7 ^cI^±0.2	9.4 ^bI^±0.1	0.0001*
**14 days**	8.5 ^aI^±0.1	11.1 ^cII^±0.1	10.6 ^bII^±0.2	0.0001*
*P*-value	1	0.004*	0.0001*	

Means with different small letters in the same row show a significant difference, while means with different capital Roman letters in the same column also show a significant difference. *Indicates a statistically significant difference (*P*-value < 0.05)

### Calcium ions release

Table 4 describes the mean values of calcium ions released by the different tested sealers at different observation times. There were significant differences between the three tested sealers in the quantity of the released calcium ions after 7 days. ADseal root canal sealer released the fewest calcium ions, while the AH Plus Bioceramic Sealer released the most calcium ions. Meanwhile, the Bio-C Sealer had intermediate results. A similar finding was detected after 14 days. Within the same sealer, there was a significant increase in the calcium ions released over time.

**Table 4:** The mean values of calcium ions release (mg/l) of the different sealers at different observation times (n = 10 per sealer)

Sealers	ADseal sealer (control)	AH Plus Bioceramic Sealer	Bio-C Sealer	*P*-value
**7 days**	0.2 ^aI^±0.1	14.1^cI^ ± 0.2	9.3^bI^ ± 0.1	0.0001*
**14 days**	1.1 ^aII^ ±0.2	28.2 ^cII^ ±0.2	23.3 ^bII^ ± 0.1	0.0001*
***P***-value	0.0001*	P = 0.0001*	P = 0.0001*	

Means with different small letters in the same row indicate a significant difference, while means with different capital Roman letters in the same column also indicate a significant difference. *Indicates a statistically significant difference (*P*-value < 0.05)

### Film thickness

The mean values of the film thickness of the different tested sealers are presented in Table 5. There were significant differences in film thickness between the three sealers. The AH Plus Bioceramic Sealer had the least film thickness, while the Bio-C Sealer had an intermediate film thickness. Meanwhile, the ADseal root canal sealer displayed the highest film thickness.

**Table 5:** Film thickness test (µm) of the different tested sealers (n = 10 per sealer)

ADseal sealer (control)	AH Plus Bioceramic Sealer	Bio-C Sealer	*P*-value
80.5 ^c^ ±1.5	20 ^a^ ±1	50 ^b^ ±3.2	P = 0.0001*

Means with different letters indicate a significant difference

### XRD results

**Fig. 1:**
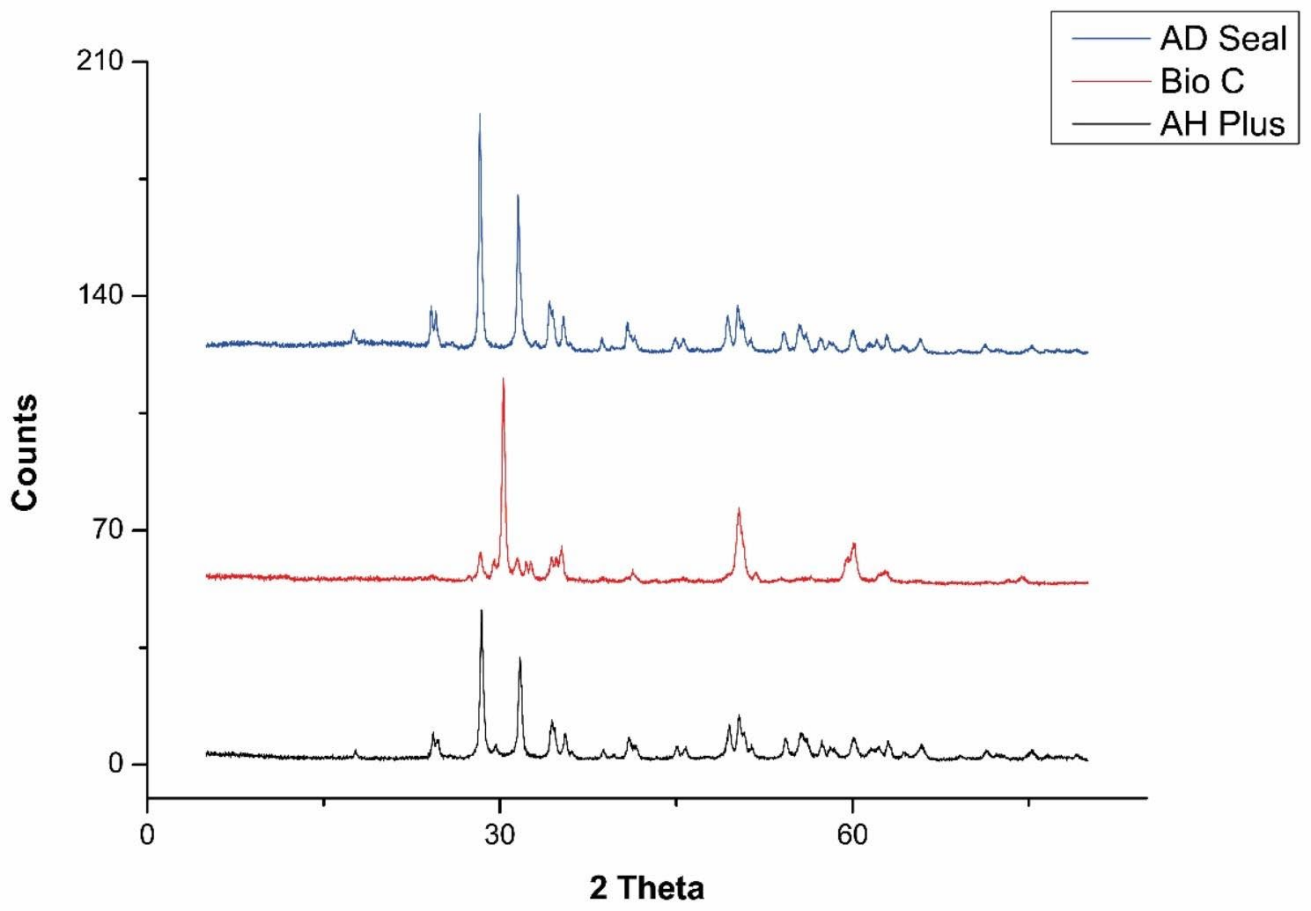
XRD patterns of AH Plus Bioceramic Sealer, Bio-C Sealer and ADseal root canal sealer (n = 1 per sealer)

The XRD analysis of sealers is represented in Fig. 1. The XRD results of AH Plus Bioceramic sealer revealed that the degree of crystallinity is about 72.5%. There are two peaks that represent zirconium oxide (monoclinic orientation) and hafnium oxide. The XRD results of Bio-C Sealer revealed that the degree of crystallinity is about 72.6%. There are two peaks that represent zirconium dioxide and zirconium oxide in tetragonal orientation. The XRD results of ADseal root canal sealer showed a degree of crystallinity of about 74%. There is one peak that represent the zirconium oxide (monoclinic orientation).

### XRF results

The XRF analysis of sealers is represented in Table 6. The major elements in ADseal root canal sealer were chlorin (57.57), silicon (9.78), zirconium (14.05), calcium (7.08), and phosphorus (6.76). However, the main elements in AH Plus Bioceramic sealer were zirconium (55.43), calcium (17.86), phosphorous (12.00), chlorin (9.64), and silicon (2.25). The main elements in Bio-C Sealer were chlorin (30.33), zirconium (37.28), calcium (14.21), phosphorous (10.75), and silicon (3.82). All tested sealers had traces of Sulfur, iron, niobium, and molybdenum.

**Table 6:** Comparison of elemental compositions of the different tested sealers by XRF analysis (n = 1 per sealer)

Components	ADseal sealer (control)	AH Plus Bioceramic Sealer	Bio-C Sealer
	Mass %	Mass %	Mass %
Si	9.78	2.25	3.82
P	6.76	12.00	10.75
Ca	7.08	17.86	14.21
S	1.89	1.42	0.57
Cl	57.57	9.64	30.33
Fe	0.89	0.14	0.61
Zn	1.35	0.12	1.13
Zr	14.05	55.43	37.28
Nb	0.27	0.10	0.49
Mo	0.29	0.23	0.67
U	0.07	0	0.14
K	0	0.46	0
Cu	0	0.10	0
Hf	0	0.03	0
Sr	0	0.04	0
Ta	0	0.12	0
Pt	0	0.05	0
Bi	0	0.01	0

## Discussion

Successful endodontic therapy is accomplished by proper sealing of the root canals with appropriate root canal filling materials. Root canal sealers are divided according to their main chemical components into zinc oxide eugenol, calcium hydroxide, glass ionomer, silicone, resin, and bioceramic-based sealers [21, 42, 43]. Bioceramic-based sealers can be categorized into calcium-silicate-based sealers, calcium hydroxide-based sealers, and calcium phosphate-based sealers [44].

Recently, a new generation of bioceramic-based root canal sealers (AH Plus Bioceramic Sealer and Bio-C Sealer) was introduced. It is assumed that their chemical behavior and surface morphology are different. Bioceramics are biocompatible, non-toxic, and chemically and thermally stable within the biological environment [21]. Bioceramic materials are categorized as bioactive or bioinert materials according to their interaction with adjacent living tissue [45].Bioinert materials, like alumina and zirconia, elicit very little reaction from the surrounding tissue and hence have no biological or physiological effect [45]. Bioactive materials, such as HA, bioactive glass, and calcium phosphates, interact with the neighboring tissue to promote the growth of new tissues [20]. However, no extensive studies of premixed calcium silicate-based root canal sealers have been conducted. ADseal root canal sealer, which is an epoxy resin-based sealer was utilized as a control sealer because it is readily available and has a reported lower solubility rate [2, 46, 47]. In addition, it is composed of calcium phosphate, comparable to calcium silicate-based sealers [2, 13].

The current in vitro study investigated the physicochemical properties (solubility, pH analysis, calcium ions release, and film thickness), crystallographic structure, and chemical elemental analysis of the newly introduced premixed calcium silicate-based root canal sealers (AH Plus Bioceramic Sealer, Bio-C Sealer), in comparison to ADseal root canal sealer.

Solubility is related to the degradation of material constituents by the dissolving actions of the surrounding fluids [35]. A high degree of solubility in root canal sealers could consistently allow gaps to be created within and between the material and the dentinal wall of the canal, thus providing a pathway for leakage from the surrounding tissues [21]. The insolubility of the root canal sealers is of great significance for successful root canal treatment through the creation of an intimate seal between the dentin wall and restoration [37]. Polymeric-based materials generally provide less solubility [48, 49].

The alkaline pH of the calcium silicate-based root canal sealers is regarded as one of their chief advantages as it leads to the formation of apatite-like deposits on the sealer surface after contact with body fluid, which enhance bioactivity and hence a strong chemical bond [50, 51]. Moreover, the alkaline pH can promote apical healing and tissue mineralization [32]. In addition, alkalinity provides bacteriostatic effects [52].

The release of calcium ions from the root canal sealers has great significance because it promotes a strong chemical bond and enhances bioactivity through the precipitation of the apatite-like layer with the dentin wall [30, 53, 54]. ICP could be used for the detection of calcium ions released in solution. Distilled water was selected as a storage medium because it has a neutral pH of 7 for easy detection of any changes in pH as well as any minor releases of calcium ions [55].

The XRD is a non-destructive test, which makes it very beneficial in several examinations [56]. It provides information about the degree of crystallinity and the crystalline composition [57]. The crystallinity of the dental materials provides stability and preservation of the structural integrity against degradation and solubility of the materials by the action of the environment [47, 58, 59]. While, XRF analysis is a rapid, and accurate test to detect chemical elemental compositions [60, 61].

The XRD analysis revealed that the crystalline part of sealers is mainly represented by the presence of zirconium oxide. The high stability of zirconium oxide limits its solubility [62]. Moreover, the tetragonal phase of zirconium may provide a higher mechanical property [63]. The XRF analysis demonstrated that the main chemical component of ADseal root canal sealer were chlorin. The presence of chlorine is explained by the presence of bisphenol-A epoxy resins in its composition [64]. However, the main chemical components of AH Plus Bioceramic Sealer and Bio-C Bioceramic Sealer were zirconium, calcium, phosphorous, and silicon. This is attributed to the fact that bioceramic sealers are calcium silicate-based, and the presence of calcium and phosphorus is related to the bioactive potentiality of the sealer [43]. The presence of bioinert zirconium is related to the stability of the materials [31]. Therefore, the higher the percent of zirconium in the sealer composition, the lower the dissolution rate [62].

According to the results of this study, the null hypothesis is rejected as significant differences are exhibited among all the tested endodontic sealers. ADseal root canal sealer (control) showed the least solubility, which may be due to the resinous nature of the epoxy-based sealer. It provides an insoluble cross-linked polymerized resin matrix that results from the polymerization of amine groups in epoxide groups [2, 65]. These findings are in accordance with Song et al. and Abu Zeid et al. [28, 66]. Meanwhile, the AH Plus Bioceramic Sealer showed intermediate solubility, which may be due to the presence of a higher amount of the crystalline stable zirconium [31], in addition to the insolubility of hafnium oxide in water [67]. It was found that ADseal root canal sealer and AH Plus Bioceramic Sealer and Bio-C Sealer achieved the minimum requirements of solubility addressed by the International Standard Organization 6876:2012, which allow a weight loss of less than 3% [68]. Bio-C Sealer showed the highest solubility rate, which may be attributed to the lower amount of zirconium than AH Plus Bioceramic Sealer. The higher solubility of calcium silicate-based sealer versus resin-based sealer is in accordance with another study conducted by Zordan-Bronzel et al. [35].

Root canal sealers that release calcium ions and have a high alkaline pH are preferred because they have an increased bioactivity [47, 69]. Though all tested sealers display high alkaline pH values (more than 8.5) at all tested time periods, different degrees of alkalinity could be observed [2].

Both AH Plus Bioceramic Sealer and Bio-C Sealer exhibit a higher degree of alkalinity, which may be related to the higher percentage of calcium and phosphorus elements in their composition compared to ADseal root canal sealer, which represents the least amount of calcium and phosphorus elements in their composition. Therefore, it might be explained by the release of calcium ions, the stimulation of mineralization, and the ability to create apatite. This is in agreement with a study by Poggio et al. [47], and Antunes et al. [70].

Both AH Plus Bioceramic Sealer and Bio-C Sealer exhibit a different degree of calcium content, which may be due to their chemical composition, which is mainly based on calcium silicates, which have a great potential for calcium and hydroxyl ions to release [70].The increase in the time of storage up to 14 days leads to more liberation of calcium ions [71]. AH Plus Bioceramic Sealer showed the highest values of calcium ion release, which may be due to their composition. This finding is in agreement with Souza et al., who displayed that the increase in calcium ions released by AH Plus Bioceramic Sealer is mainly related to their major components, which are oxygen, calcium, phosphorus, and zirconium [72].

The film thickness of the root canal sealers is an essential factor because a thin film thickness enhances the wettability of the materials on the canal wall, providing appropriate sealing [11]. A decrease in the film thickness of root canal sealer influences the sealing of a root canal with a minimum microleakage [31]. Film thickness is affected mainly by the compositional constituents and particle size [12]. The reduced film thickness of AH Plus Bioceramic Sealer may be attributed to its reduced particle size; this finding coincides with the manufacturer’s claims. Moreover, the presence of a higher amount of zirconium could improve the flow and reduce the film thickness of the sealer [31]. Despite the intermediate film thickness of ADseal root canal sealer and the highest film thickness of Bio-C Sealer, they both met the ISO recommendation of 50 μm for sealer film thickness [39]. The lack of in vivo experiments, short-term assessment, and the difference in the surrounding conditions from the clinical situation may be considered limitations of the current study. Moreover, further studies are recommended to examine the particle size of the root canal sealers. In addition, other physical investigations such as viscosity, setting time, and radiopacity are recommended to be evaluated.

## Conclusions

Our findings suggest that the chemical composition and the degree of crystallinity greatly affect the solubility of the root canal sealers. Calcium ions released are responsible for increasing pH values. Furthermore, both AH Plus Bioceramic Sealer and Bio-C Sealer could be used as a potential alternative to conventional resin-based root canal sealers regarding its solubility, alkalinity, calcium ion release, and film thickness.

## Data Availability

The data that support the findings of this study are available from the corresponding author upon reasonable request.

## Abbreviations

XRD: X-ray diffraction analysis
XRF: X-ray fluorescence
Rh: Rhodium
MREC: The Medical Research Ethical Committee
NRC: National Research Centre
ART: Attenuated total reflection
JCPDS: Joint Committee on Powder Diffraction Standard
ICP-OES: Inductively Coupled Plasma optical emission spectroscopy
ISO: International Standard Organization
Si: Silicon
P: Phosphorus
Ca: Calcium
S: Sulfur
Cl: Chlorine
Fe: Iron
Zn: Zinc
Zr: Zirconium
Nb: Niobium
Mo: Molybdenum
U: Uranium
K: Potassium
Cu: Copper
Hf: Hafnium
Sr: Strontium
Ta: Tantalum
Pt: Platinum
Bi: Bismuth
